# Experimental Investigation of Electrical Conductivity and Permittivity of SC-TiO_**2**_-EG Nanofluids

**DOI:** 10.1186/s11671-016-1590-7

**Published:** 2016-08-24

**Authors:** Jacek Fal, Adriana Barylyak, Khrystyna Besaha, Yaroslav V. Bobitski, Marian Cholewa, Izabela Zawlik, Kamil Szmuc, Józef Cebulski, Gaweł żyła

**Affiliations:** 1 Department of Physics and Medical Engineering, Rzeszów University of Technology, Rzeszów, Poland; 2 Danylo Halytskyy Lviv National Medical University, Lviv, Ukraine; 3 Department of Silicate Engineering, Lviv Polytechnic National University, Lviv, Ukraine; 4 Department of Photonics, Lviv Polytechnic National University, Lviv, Ukraine; 5 Department of Biophysics, Faculty of Mathematics and Natural Sciences, University of Rzeszów, Rzeszów, Poland; 6 Laboratory of Molecular Biology, Centre for Innovative Research in Medical and Natural Sciences, Faculty of Medicine, University of Rzeszów, Rzeszów, Poland; 7 Department of Experimental Physics, Faculty of Mathematics and Natural Sciences, University of Rzeszów, Rzeszów, Poland; 8 Center for Innovation and Transfer of Natural Sciences and Engineering Knowledge, University of Rzeszów, Rzeszów, Poland; 9 Department of Genetics, Chair of Molecular Medicine, Faculty of Medicine, University of Rzeszów, Rzeszów, Poland

**Keywords:** Dielectric properties, Nanofluids, Titanium oxide

## Abstract

The paper presents experimental studies of dielectric properties of nanofluids based on ethylene glycol and SC-TiO_2_ nanoparticles with average size of 15–40 nm with various mass concentrations. The dielectric permittivity both real part and imaginary part as a function of temperature and frequency were measured. Also, dependence ac conductivity on frequency, temperature, and mass concentration were investigated. Based on the curves of ac conductivity, dc conductivity was calculated, and 400 % enhancement in dc conductivity was exposed.

## Background

In an era of continuous technology development, scientists and engineers constantly strive to achieve better and better results in various fields of science and technology. Regardless of whether it is an increase in energy efficiency or miniaturization of devices or medical applications, many of the researchers believe that the nanomaterials are the future of technique and industry. One of the most commonly studied group of nanomaterials are nanofluids. This name was established by Choi [1] in 1995. Nanofluids are identified as a suspension of solid particles with nanometric size (1–100 nm) in based fluids. As base fluids are usually used distilled water, oils, or alcohols, e.g. ethylene glycol. So much interests of nanofluids is due to their unusual thermophysical properties, completely different from the same materials in macro scale.

Nanofluids have a higher thermal conductivity, higher electrical conductivity, and absorption of light. Thanks to all of these properties, nanofluids have a huge potential in industry. The most frequently mentioned applications are all processes where there is a need to discharge large amounts of heat, for example: double pipe and multichannel heat exchanger [2–6], engine cooling, cooling of electronics, chillers, domestic refrigerator [7, 8], solar water heating [9–11], cooling, and heating in buildings [12].

Due to many of potential applications, nanofluids are extensively studied in many fields of science. The rheological properties of nanofluids were investigated by many researchers and were published a few review papers [13–15].

TiO_2_–EG nanofluids have been examined for rheological properties by Cabaleiro et al. [16], and they presented that this material exhibit a non-Newtonian shear-thinning nature, on the other hand Khedkar et al. [17] classified this material as Newtonian fluid. This shows that the physical properties of nanofluids require further experimental studies. Similar nature was presented by Tseng and Lin [18] for water-based TiO_2_ nanofluids. This material is extensively studied because of its interesting thermal properties [19–22]. Khedkar et. al. [17] presented results of experimental investigations and theoretical determination of thermal conductivity of TiO_2_–ethylene glycol nanofluids. They confirmed that thermal conductivity in these nanofluids increase linearly with volume concentration of nanoparticles.

Increase in thermal conductivity of TiO_2_ nanofluids (ethylene glycol water mixture) was also presented by Reddy and Rao [23].

Also, thermal properties of nanofluids were widely investigated both in experimental and theoretical field. Leong et al. [24] studied thermal properties of multiwalled carbon nanotube water based nanofluids. Ahammed et al. [25] investigated thermal conductivity of graphene–water nanofluid for various concentrations and temperatures. Palabiyik et al. [26] conducted experimental measurements of thermal conductivity of aluminium oxide (Al_2_O_3_) and titanium oxide (TiO_2_) propylene glycol-based nanofluids in temperature range form 293.15–353.15 K and reported non-linear increase of thermal conductivity with increasing in concentration. Murshed et al. [27] also studied TiO_2_ nanoparticle suspension in base fluid (water), and they also reported enhancement of thermal conductivity with increase in volume fraction of nanoparticles.

The electrical conductivity and dielectric properties are likewise investigated, but in comparison to the rheological or thermal properties, they are less popular among researchers. The electrical conductivity of silver and aluminium oxide nanofluids was studied by Maddah et al. [28]. They reported significant increase in electrical conductivity as compared to base fluid which was water. Minea et al. [29] investigated electrical properties of aluminium oxides-water nanofluids too. They conducted measurements in temperature range from 298.15 to 343.15 K for various volume concentrations (1–4 %) and have found growth in electrical conductivity with increase in volume fraction and temperature. The maximum enhancement was achieved for 4 % volume concentration of nanoparticles in water at a temperature 333.15 K and it was 390.11 %.

The huge enhancement in electrical conductivity (almost 60000 % for 20 wt. % at 298.15 K) was observed for AlN nanoparticles suspended in ethylene glycol as reported in Ref. [30]. Adio et al. [31] conducted research on electrical conductivity of MgO particles suspended in ethylene glycol. They reported enhancement in electrical conductivity more than 3000 %.

Angayarkanni and Philip [32] showed a 5218, 4366, 3142, and 8876 % increase in electrical conductivity, respectively, for *α*-Al_2_O_3_, SiO _2_,*γ*-Al_2_O_3_, TiO_2_ water-based nanofluids. They explain such a large increase as an effect of double electric layer surrounding each particle and particle size effect. Nanosuspension of TiO_2_ particles in water-based fluid were also investigated by Sikdar et al. [33]. As in previous cases, Sikdar et al. found an increase in electrical conductivity with increase in volume concentrations, but they also observed decrease in rate of increase with increasing in volume concentrations.

Some researchers also investigate dielectric properties of nanofluids such as permittivity, ac conductivity, or relaxation time in various types of nanofluids [34–39].

Dielectric properties of TiO_2_ nanofluids were studied by Subramaniyan et al. [40] for three TiO_2_ nanofluids with ethylene glycol, propylene glycol and water-based fluids for two frequencies (10^4^ and 10^7^ Hz) at 293.15 K. They reported increase in dielectric constant for all samples and the largest growth was observed for water-based nanofluid. These studies provide information in a narrow range of temperature and frequencies. To better understand phenomena occurring in nanofluids based on TiO_2_ nanoparticles, further experiments are needed in a wider range of temperatures and frequencies, and this is the main scope of this paper.

## Nanoparticle preparation and characterization

Yellow powder of SC-TiO_2_ nanoparticles was synthesized using a solid phase method. The 10.4 g of metatitanic acid and 3.6 g of thiourea (provided by Wako Pure Chemical Industry) were triturated in an agate mortar to obtain a homogeneous mass, which was heated up at 773.15 K per 1 h. Based on our own experiments and published literature, thermolysis process takes place in the following steps: (1) endothermal process with a maximum at 408.15 K related to a dehydration methatitanic acid, (2) endothermic effects with a maximums of 448.15 and 468.15 K connected to the melting of thiourea, (3) exothermal process at 513.15 K linked to crystallisation of anatase with in presence of thiourea and simultaneous thermal degradation to the above described gaseous products, (4) exothermal processes with a maximums at 588.15 and 688.15 K showing the thermal oxidation of the organic residues, sulphur and continued crystallization of anatase. The loss of weight of thiourea and methatitanic acid on heating is completed at a temperature of 688.15 K, but optimum temperature for the sintering of sample is 773.15 K because in lower temperature, sulphur mixture could interact with anatase. The total weight loss in the temperature range 273.15–773.15 K is 9.4 wt.%.

**Fig. 1:**
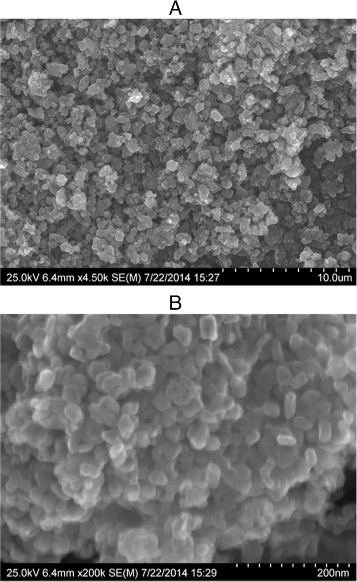
SEM pictures of obtained SC-TiO_2_ nanoparticles, **a** magnification 4.5k, **b** magnification 200k

Surface morphology of SC-TiO_2_ powder was characterized by high–resolution scanning electron microscope Hitachi S-4800 II. SEM imaging showed micrometer-sized randomly distributed crystal aggregates, in the range of 5–15 *μ*m (Fig. 1a).

The higher magnification imaging (Fig. 1b) revealed that the observed crystal aggregates consist of many 15–40 nm sized SC-TiO_2_ nanoparticles which is almost 10 times smaller than in pure TiO_2_ powder.

The particle size and its distribution measured by a laser dynamic light scattering were much larger in comparison to obtained by microscopic methods. It happens because nanoparticles after doping by sulphur have a tendency to agglomerate into bigger aggregates, which are analyzed by DLS as complexes, not like a separate nanoparticles.

According to the XRD analysis performed to obtain phase composition, SC-TiO_2_ powder contains only one crystalline phase—the tetragonal modification of TiO_2_-anatase. In comparison to the pure TiO_2_ sample, a slight c lattice parameter and volume increase due to incorporation of additional elements. The phase identification of doped TiO_2_ was done by Siemens D5000 diffractometer in reflection mode with Cu target K *α* radiation. XRD patterns were recorded in the 2 *θ* range of [15; 100°] with a step size of 0.02° and a stay time of 1 s/step.

UV-VIS study performed by Evolution 300 UV-VIS spectrophotometer (Thermo Scientific) in the range from 300 to 800 nm suggests that SC-TiO_2_ nanoparticles got improved photocatalytic properties in comparison to pure TiO_2_. Recorded UV-VIS absorption spectra showed that the material is catalytically active in UV region and additional in some parts of VIS spectrum.

Raman spectroscopy was performed by Smart Raman DXR (Thermo Scientific) spectrometer. The semiconductor laser of 12 mW power and 780 nm wavelength was used as a light source. The obtained data confirmed the anatase crystal structure of doped TiO_2_ nanoparticles. The EPR analysis revealed that the sensitization process of the nanopowders doped by sulfur and carbon is a result of the formation of additional oxygen vacancies into TiO_2_ structure, which are essential for the photocatalytic activity of the material. According to XPS, carbon is in the form of elemental carbon compounds (Eb = 285.0 eV), and substances having a C-O bond (shoulder near 287 eV and a peak at 289 eV), which are adsorbed on the particle surface. Oxygen is included in the composition of oxides (Eb = 530.6 eV), and the carbon containing adsorbate (shoulder at 532 eV). The binding energy of titanium (459.0 ± 0.3 eV) and sulfur (169.0 ± 0.3 eV) core shell for S-TiO_2_ powders correspond to established data for TiO_2_ and MeSO_4_ compounds, respectively. In particular, sulphur is found exclusively in the oxidation state +6. The XPS results showed that sulphur and carbon are concentrated predominantly in the upper (periphery) layer of the nanoparticle. The quantification of the chemical elements detected by XPS analysis displays 2.8 % sulphur and 30.3 % carbon on the TiO_2_ nanoparticles surface, while the concentration of both elements drops to 0.17 % for S and for C detected in the particles volume. A detailed description of the preparation of nanoparticles is presented in Ref. [41].

## Sample preparation

The nanofluids used in experiment were prepared using a two-step method. First, an appropriate amount of nanoparticles and ethylene glycol has been weighed with the analytical balance WAS 220/X (Radwag, Radom, Poland). Then, samples were mechanically agitated for 30 min using Genius 3 Vortex (IKA, Staufen, Germany). The last step of preparation of samples was sonication in ultrasonic bath Emmi 60 HC (EMAG, Moerfelden-Walldorf, Germany) in order to break up the agglomerates remaining after the mechanical stirring. The time for sonication was 200 min. An ultrasound that we used has a power of about 350 W, and is equipped in six ultrawave generators with frequency about 45 kHz.

## Methods

The dielectric properties of SC-TiO_2_-EG nanofluids were measured using BroadBand Dielectric Spectrometers Novocontrol Concept 80 System with temperature control system Quatro Cryosystem (NOVOCONTROL Technologies GmbH & Co. KG, Montabaur, Germany). This measuring stand allows to conduct measurements in broad range of frequencies and temperatures. Measurements were carried out in frequency range from 0.01 Hz to 10 MHz in 51 steps. Temperature was changed from 273.15 to 333.15 K with 5 K step, and stabilized at least 15 min before measurements. The samples were placed between two brass electrodes with diameter of 40 mm. The gap between electrodes was set by teflon fabric with 0.16 mm. The standard deviation of measurements has been determined on the basis of 10 measurements of dielectric constant of glycerine for 293.15, 298.15, 313.15, and 333.15 K. The results were compared with data published by Glycerine Producers’ Association [42]. The maximum standard deviation of measurements was observed at 333.15 K and it was 6 %, and the minimum standard deviation of measurements was observed at 293.15 K and it was 5.6 %.

## Results and discussion

**Fig. 2:**
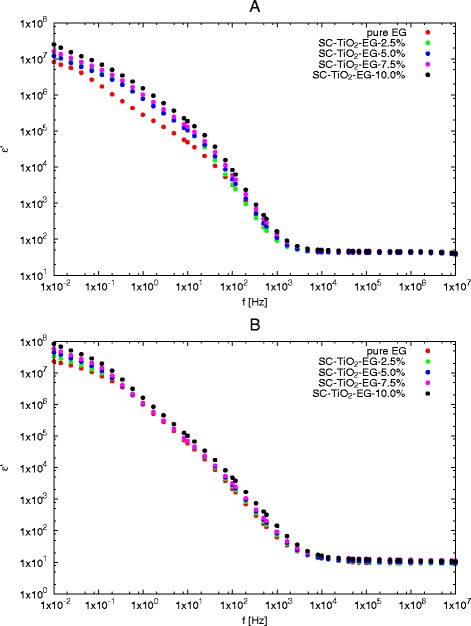
Dielectric constant as a function of frequency for various mass concentrations at different temperatures: **a** 273.15 K, **b** 333.15 K

The variation of dielectric constant (*ε*^′^) with frequency for SC-TiO_2_-EG nanofluids with various mass concentrations at two temperatures are presented in Fig. 2. It is obvious that dielectric constant is frequency-dependent in some regions depending on temperature. In frequency ranges below 10 kHz, it can be observed decrease in dielectric constant with increase in frequency which is connected with relaxation process in nanofluids. Above this frequency (10 kHz), *ε*^′^ is almost constant despite the increase in frequency. Also, effect of particle concentrations is clearly noticeable in low frequencies and low temperatures (273.15 K). Whereas at temperature of 333.15 K impact of mass concentration seems to fade away; only below 1 Hz is still clearly visible.

**Fig. 3:**
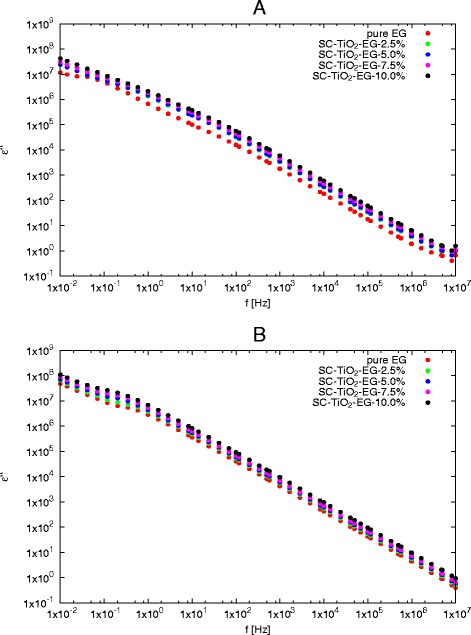
Loss factor as a function of frequency for various mass concentrations at different temperatures **a** 273.15 K, **b** 333.15 K

Figure 3 shows frequency dependence of loss factor for various mass concentrations at minimum and maximum tested temperatures. Analysing data presented in Fig. 3, it can be found that *ε*^′′^ decreases with increase in frequency almost linear in whole range of frequency and temperatures. At low frequency range (below 1 Hz), a slight deviation is visible.

**Fig. 4:**
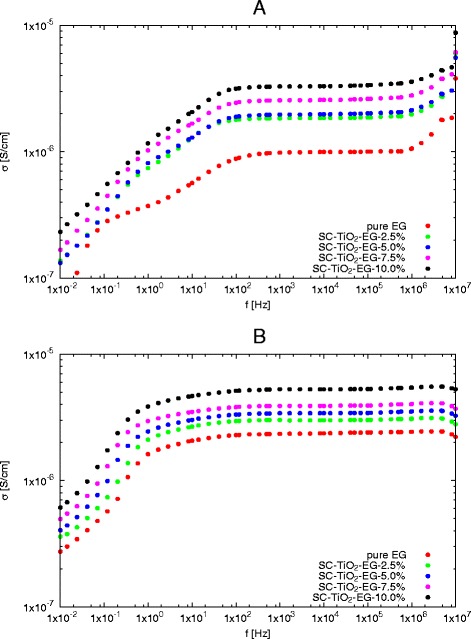
AC conductivity as a function of frequency for various mass concentrations at different temperatures **a** 273.15 K, **b** 333.15 K

**Fig. 5:**
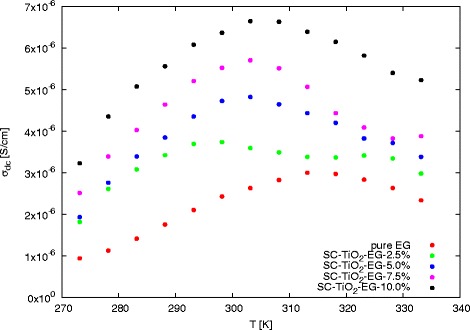
DC conductivity as function of temperature for various mass concentrations

The changes of ac conductivity (*σ*_*ac*_) as function of frequency for nanofluids with various mass concentrations were show in Fig. 4. Based on these data, it is obvious that frequency has strong impact on ac conductivity in frequency below 100 Hz at 273.15 K. For higher temperatures this border is shifted toward lower frequencies, at 333.15 K it is about 10 Hz. Analysing the ac conductivity curves can be observed plateaus between 100 Hz and 1 MHz for all concentrations of nanoparticles in ethylene glycol. The width of plateaus does not depend on mass concentration of nanoparticles, but with increasing in temperature increase width of plateaus in *σ*_*ac*_, and at 333.15 K is between 10 Hz and 10 MHz. The similar effect was observed by Dey et al. [43] for polyaniline-TiO_2_ nanocomposites. The constant value of conductivity in this frequency regions corresponds to dc conductivity (*σ*_*dc*_) [43]. The values of dc conductivity for SC-TiO_2_-EG nanofluids were calculated as the average of ac conductivity in frequency range from 100 Hz to 1 MHz and summarized in Table 1. The graphical representation of dc conductivity as function of temperature for various mass concentrations was shown in Fig. 5.

**Table 1:** DC conductivity and enhancement dc conductivity of SC-TiO_2_-EG nanofluids with various mass concentrations and temperatures

T [K]	*σ*_*dc*_×10^−6^[*S*/*c* *m*]					*σ*_*dc*_/*σ*_0_[−]			
	0 wt.%	5 wt.%	10 wt.%	15 wt.%	20 wt.%	5 wt.%	10 wt.%	15 wt.%	20 wt.%
273.15	0.95	1.82	1.94	2.52	3.23	1.92	2.04	2.65	3.41
278.15	1.13	2.62	2.76	3.39	4.35	2.31	2.44	3.00	3.85
283.15	1.42	3.08	3.39	4.03	5.07	2.18	2.40	2.85	3.58
288.15	1.76	3.43	3.85	4.64	5.57	1.95	2.19	2.64	3.16
293.15	2.11	3.70	4.35	5.21	6.08	1.75	2.06	2.47	2.88
298.15	2.43	3.74	4.73	5.53	6.37	1.54	1.94	2.27	2.62
303.15	2.64	3.60	4.83	5.71	6.65	1.36	1.83	2.16	2.52
308.15	2.83	3.50	4.65	5.52	6.63	1.24	1.64	1.95	2.35
313.15	3.00	3.38	4.44	5.07	6.40	1.13	1.48	1.69	2.13
318.15	2.98	3.38	4.20	4.44	6.15	1.13	1.41	1.49	2.07
323.15	2.84	3.42	3.83	4.09	5.82	1.20	1.35	1.44	2.05
328.15	2.63	3.35	3.72	3.83	5.40	1.27	1.41	1.45	2.05
333.15	2.34	2.99	3.39	3.88	5.23	1.28	1.45	1.66	2.23

**Fig. 6:**
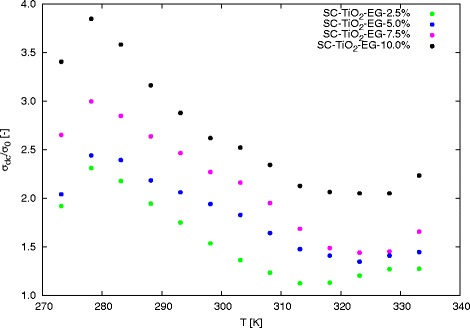
Enhancement in dc conductivity as function of temperature for various mass concentrations

As can be seen, increase in temperature cause changes in dc conductivity. Increase in dc conductivity can be observed at temperatures below 305 K for all concentrations of nanoparticles in base fluid. Above this temperature, *σ*_*dc*_ decrease with increase in temperature. This effect can be attributed to agglomeration of nanoparticles and sedimentation, caused by intensification of Brownian motion in nanofluids. Second part of Table 1 contain enhancement in dc conductivity at various temperature, calculated as ratio between dc conductivity of nanofluids and dc conductivity of pure base fluid. Graphical interpretation of enhancement in conductivity was presented in Fig. 6. The maximum enhancement was achieve for 20 wt.% at 278.15 K and it was almost 400 %. Initially, enhancement increase with increase in temperature for all mass concentrations, but above the 278.15 K can be observed decrease in enhancement of dc conductivity. The comparable behaviour was observed by Dong et al. [44] for AlN nanoparticles suspended in transformer oil. The initial increase and subsequent decrease in enhancement was also exposed by Shirazi et al. [45] for nitrogen doped activated carbon/graphene nanofluids.

**Fig. 7:**
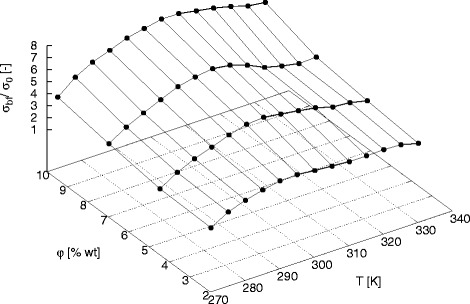
Enhancement in dc conductivity as function of mass concentration for various temperatures

The dependence of electrical conductivity enhancement on mass concentration for various temperatures was presented in Fig. 7. For each temperature can be observed almost linear increase in electrical conductivity enhancement with increase in mass concentration of SC-TiO_2_ nanoparticles in base fluid. Linear growth in electrical conductivity enhancement with increase in mass concentration was also observed for aluminium oxide water nanofluids [32, 46] and silicon oxide and titanium oxide water nanofluids [32]. The significant electrical conductivity enhancement is caused, among others, by creating a double electrical layer around nanoparticles and creating conduction paths in higher concentrations of nanoparticles in base fluid [47, 48].

## Conclusions

The complex permittivity (*ε*^′^,*ε*^′′^), ac conductivity (*σ*_*ac*_) and dc conductivity (*σ*_*dc*_) in broad frequency range (0.01 Hz–10 MHz) and temperatures from 273.15 to 333.15 K for ethylene glycol with various mass concentrations of titanium oxide were investigated. Results indicate that frequency have strong influence on real part of complex permittivity in range below 10 kHz. Also, imaginary part of permittivity is strongly linked with the frequency in whole range. Based on the plateaus in ac conductivity, the dc conductivity was computed, and found almost 400 % increase in electrical conductivity for 20 wt.% at 278.15 K.
